# New Echinoderm-Crab Epibiotic Associations from the Coastal Barents Sea

**DOI:** 10.3390/ani11030917

**Published:** 2021-03-23

**Authors:** Alexander G. Dvoretsky, Vladimir G. Dvoretsky

**Affiliations:** Murmansk Marine Biological Institute (MMBI), 183010 Murmansk, Russia; v-dvoretsky@yandex.ru

**Keywords:** epibiosis, red king crab, *Paralithodes camtschaticus*, common starfish, brittle star, Atlantic sea cucumber, green sea urchin, Barents Sea

## Abstract

**Simple Summary:**

New biodiversity records are important for expanding our knowledge about the symbiotic associations of the commercially important red king crab. This species was introduced into the Barents Sea, and now its population supports a viable fishery in the area. There are only a few reports regarding epibiotic relationships between echinoderms and marine crabs in general and the red king crab in particular. In our paper, we provide new data on the occurrence of the common starfish, Atlantic sea cucumber, green sea urchin, and brittle star on the invasive red king crab in the Barents Sea. The associations between echinoderms and red king crabs could have important ecological implications and provides an interesting example of how a prey species can avoid death by infesting its predator.

**Abstract:**

During diving surveys for a Russian research project that monitored introduced species, red king crabs (*Paralithodes camtschaticus*) were collected at a coastal site of the Barents Sea to study the structure and dynamics of this species. Sampling of the organisms colonizing the crabs was part of this research project. For the first time, the presence of relatively large specimens of the common starfish *Asterias rubens* as epibionts of *P. camtschaticus* was observed in July 2010, 2018, and 2019. In 2010 and 2019, we also found three other echinoderm species (the Atlantic sea cucumber *Cucumaria frondosa*, the green sea urchin *Strongylocentrotus droebachiensis,* and the brittle star *Ophiura sarsii*). These findings add to the current list of associated species on king crabs not only in the Barents Sea but also in native areas of this host. Red king crabs have been documented as predators for these echinoderm species, and our records show additional possible interactions between king crabs and echinoderms in this region. More likely, the epibiotic lifestyle allows these echinoderms to avoid predation from red king crabs. There are no potential disadvantages derived by red king crabs through their relationships with the echinoderm epibionts due to low occurrences of these associations. We suggest no negative effects for the local red king crab population and populations of other commercial species in the Barents Sea.

## 1. Introduction

The red king crab, *Paralithodes camtschaticus* (Tilesius, 1815) is one of the few large, higher trophic level marine crustaceans in the World Ocean. It is a highly valued delicacy on the international market and currently contributes significantly to the income from fisheries in the regions where it is harvested, i.e., Russia, Norway, and the USA [1]. Red king crab was introduced to the Barents Sea from the northern Pacific (Sea of Okhotsk and Sea of Japan) in the 1960s to establish a new commercial fishery [2,3]. Since then, this invasive species has spread west along the Norwegian coast and northeast of the Kola Peninsula from the original area of introduction, Kola Bay, Russia. This introduction had no negative economic and fishery impact in Russian waters of the Barents Sea [3]. In Russian waters of the Barents Sea, red king crabs occur from shallow (3 m) to deep waters (335 m), at −0.8 °C to +8.5 °C [3]. In spring, they form mating aggregations at coastal sites. In autumn, red king crabs segregate by sex, with males and females forming aggregations in deep and shallow waters [1]. Red king crabs are high-level predators feeding on the most abundant benthic organisms including bivalve and gastropod mollusks, polychaetes, and echinoderms. In areas with multispecies fishing, they predominantly feed on fish offal [1,3]. The main predators of *P. camtschaticus* in the Barents Sea are cod, wolffish, and skates [2]. The crab became a member of the local benthic system and the stocks of *P. camtschaticus* are regulated both by environmental and anthropogenic factors such as trophic interactions, climate fluctuations, and fishery [1,2,4,5,6,7]. In 2018, 2019, and 2020, total abundances of *P. camtschaticus* were estimated to be 38, 44, and 58 million crabs [8] and landings reached 9187, 9836 and 10,820 metric tons, respectively [8,9].

Epibiosis is a widely distributed phenomenon in the marine environment. It is defined as a facultative association of two organisms: the basibiont (or host) and the epibiont. The epibiont is attached to the surface of a living substratum, while the host lodges and constitutes support for its epibiont [10]. The exoskeleton and gills of *P. camtschaticus* are colonized by several epibionts/symbionts including hydrozoans, nemerteans, polychaete worms, fish leeches, bivalve and gastropod mollusks, amphipods, cirripedians, copepods, and bryozoans [11,12,13,14]. The echinoderm epibiosis on king crabs has been poorly documented, but predator-prey interactions between *P. camtschaticus* and echinoderms are well documented [15,16].

Coastal sites of the Barents Sea play a crucial role in the growth and recruitment of the local red king crab population [7]. For this reason, since 2003, monitoring of the *P. camtschaticus* population has been conducted by specialists from the Murmansk Marine Biological Institute (MMBI) in coastal waters of the Kola Peninsula [4,7,13,17,18]. Regular studies of symbiotic associations of non-indigenous species are important because they allow the tracking of the establishment and adaptation processes of such alien host species in new places of their distribution and could help evaluate the impacts of these crabs on local communities [19,20,21]. In addition, such studies could provide new information on the biodiversity of targeted areas and detect changes in local communities associated with human activities and climate shifts [20,22].

The aim of this study is to document new evidence of an epibiosis between common echinoderms and red king crab living in the Barents Sea.

## 2. Materials and Methods

### 2.1. Study Area

**Figure 1:**
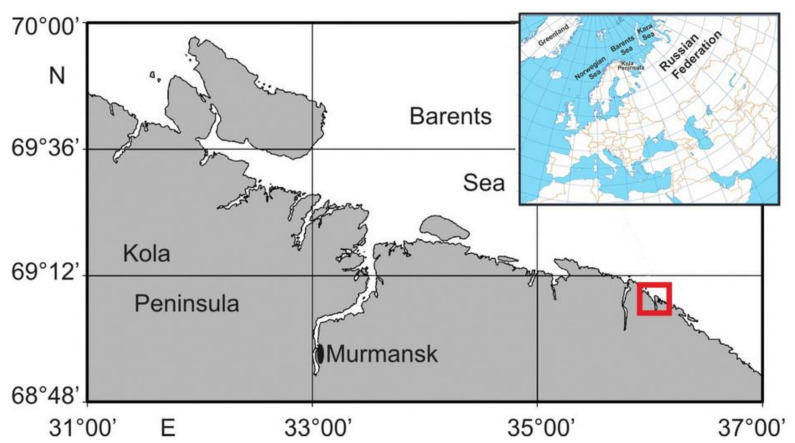
Location of the study area (Dalnezelenetskaya Bay, 69°07′ N, 36°05′ E).

Dalnezelenetskaya Bay is a semi-closed small gulf with 5 islands separating the area of this bay from the open sea (Figure 1).

This coastal site is almost square (2 × 2 km) with a total area of 2.23 km^2^ size [7]. The maximum depths are registered in the western part of the area. Mean depth is ca. 7 m. Tidal levels are high enough (3–4 m) to ensure intensive water exchange between the inner part of the bay and the open sea.

The lowest temperature level in the surface layer (0.7 °C) occurs in February and the maximum (9.7 °C) in August. Salinity minimum (32.2 psu) is associated with high input of meltwater and it is registered in May. In autumn and winter, salinity is stable accounting for 34 psu [7,23]. The minimum level of dissolved oxygen (94%) is registered in December, the maximum (124%) in May [23]. In July, water temperature is 9.1 °C, salinity is 32.7 psu, and the concentration of dissolved oxygen is 104.4% [23].

### 2.2. Sampling and Processing

Diving works were undertaken in July 2010, 2018, and 2019. The crabs colonized by large epibiont species were placed in individual bags, other specimens were transferred to the laboratory without sorting. The crabs collected by SCUBA were transferred on the coast immediately after capture. A total of 365 red king crabs (*n* = 133 in 2010, *n* = 141 in 2018 and *n* = 91 in 2019) were collected at depths from 5 to 40 m.

Each crab was examined for the sex (according to the form of the abdominal flaps), carapace length (CL, the greatest straight-line distance across the carapace from the posterior margin of the right eye orbit to the medio-posterior margin of the carapace), and shell condition according to Donaldson and Byersdorfer [24].

Crabs were examined for associated species in the laboratory in Dalnezelenetskaya Bay by eye, according to previous studies [13]. The body of red king crabs was divided into five sections: carapace, limbs (walking legs and claws), abdomen, mouthparts, and gills. All epibionts were fixed in 4% formalin and then identified in the laboratory in Murmansk using a stereomicroscope MBS-10.

We used the two standard indices of infestation [25]: prevalence (the proportion of crabs colonized by an associate species, %) and intensity (the number of the associate specimens per colonized crab). Mean values are presented with standard deviations.

The taxonomic nomenclature follows the nomenclature according to WoRMS [26].

## 3. Results

**Figure 2:**
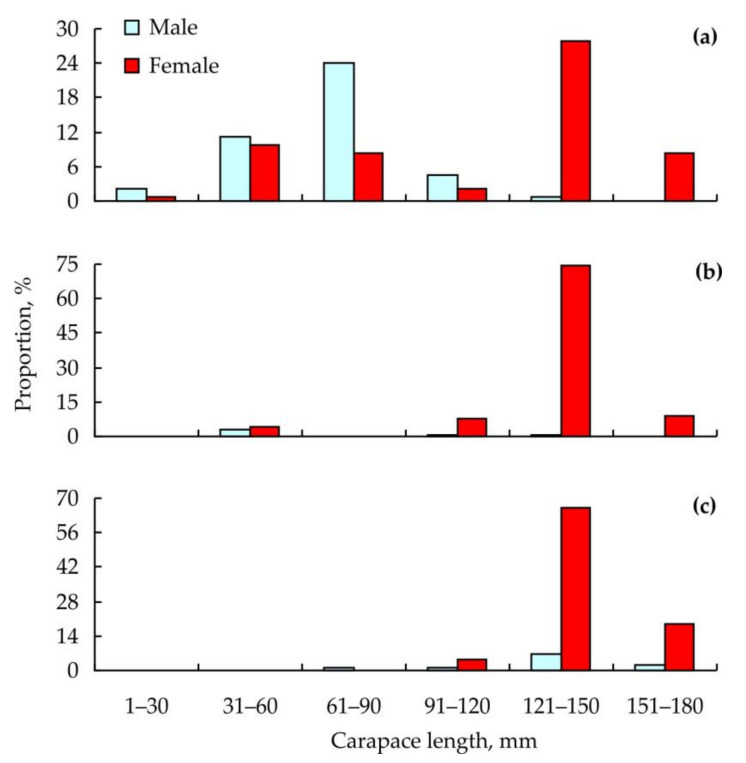
Size-frequency distributions of red king crabs collected for epibionts in Dalnezelenetskaya Bay in 2010 (**a**), 2018 (**b**), and 2019 (**c**).

In 2010, mean carapace length was 71.7 ± 21.6 mm in males (*n* = 57) and 113.7 ± 38.7 mm in females (*n* = 76). In 2018 and 2019, these values were 79.7 ± 13.6 mm (males, *n* = 6) and 133.5 ± 1.8 mm (females, *n* = 135) and 134.5 ± 8.6 mm (males, *n* = 10) and 140.8 ± 1.2 m (females, *n* = 81), respectively. Size-frequency distributions of the crabs are presented in Figure 2.

In total, 39, 34, and 52 taxa of associated organisms were found on the red king crabs in 2010, 2018, and 2019, respectively. In 2010, the maximum prevalences and mean intensities of infestation were registered for amphipods *Ischyrocerus* spp. (32–68%, 2.8–45.9 ind. per crab), copepods *Tisbe* spp. (36%, 19.5 ind. per crab) and cirripedians *Balanus* spp. (5–25%, 2.4–3.9 ind. per crab). In 2018, the prevalences/mean intensities of the amphipods and copepods were 22–92%/7.5–68.0 ind. per crab and 17–91%/2.8–16.5 ind. per crab, respectively. In 2019, these indices ranged from 15–100%/6.9–102.5 ind. per crab and 31–99%/4.9–31.0 ind. per crab.

**Figure 3:**
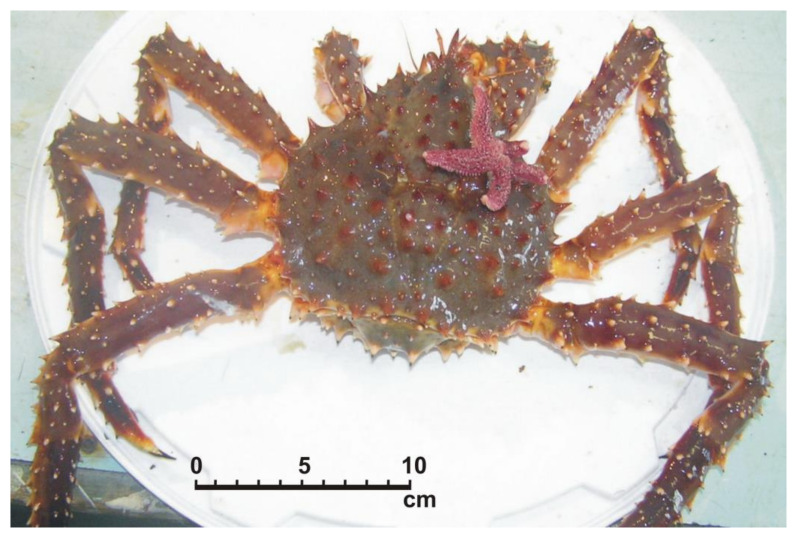
A specimen of the common starfish *Asterias rubens* on the carapace of a male red king crab *Paralithodes camtschaticus*.

On 6 July 2010, a male red king crab (new shell, CL 95 mm, weight 665 g) harboring a single specimen of *Asterias rubens* (Linnaeus, 1758) on its body was caught by a diver in the study area (69°07′42.5″ N, 36°05′11.4″ E) at a depth of 20 m. Although this epibiont was firmly attached to the carapace of its host, the crab was placed in an individual bag according to our sampling protocol and then transferred to the laboratory, where it was photographed (Figure 3).

The starfish was removed from the host carapace. Weight of this epibiont was 42 g, arm length (AL) 29 mm. The second starfish specimen (weight 55 g, AL 34 mm) was registered on a male red king crab (new shell, CL 126 mm, weight 1194 g) collected in the area at 13 m on July 08, 2018, and the final finding was recorded on July 09, 2019, when our divers captured an adult male crab (new shell, CL 136.4 mm, weight 2115 g) colonized by a single sea star weighing 41 g. Both epibionts had the same localization as we recorded in 2010. Thus, the prevalences of *A. rubens* were 0.7, 0.8, and 1.1% in 2010, 2018, and 2019, respectively.

In addition to *A. rubens*, three other epibiotic echinoderms, the green sea urchin *Strongylocentrotus droebachiensis* (O. F. Muller, 1776) (diameter 8 mm), the brittle star *Ophiura sarsii* Lütken, 1855 (disc diameter 11 mm) and the Atlantic sea cucumber *Cucumaria frondosa* (Gunner, 1767) (body length 12 mm), were observed under a stereomicroscope in the samples in 2010 and 2019. The first epibiont was found on the carapace of an immature male crab (new shell, CL 60.0 mm, weight 159.5 mm), the second species was found on the carapace of a large male (old shell, CL 165 mm, weight 3443 g) and the third echinoderm occurred on the abdomen of an egg-bearing female (new shell, CL 155.6 mm, weight 2385 g).

In 2010, 2018, and 2019, the prevalences of echinoderms were 5.3, 16.7, and 10% on males and 0, 0, and 1.2% on females.

The data on crab epibionts found together with these echinoderms are summarized in Table 1.

## 4. Discussion

There are only a few reports regarding associations between echinoderms and crabs. Stachowitsch [27] reported the feather star *Antedon mediterranea* (Lamarck, 1816), the sea cucumber *Ocnus planci* (Brandt, 1835) (cited as *Cucumaria planci*), and the brittle star *Ophiothrix quinquemaculata* (Delle Chiaje, 1828) as epibionts of the hermit crabs *Paguristes eremita* (Linnaeus, 1767) and *Pagurus cuanensis* Bell, 1845 from the North Adriatic Sea (Gulf of Trieste). The last epibiont was also found on the red reef hermit crab *Dardanus arrosor* (Herbst, 1796) in the Mediterranean Sea [28]. The brittle stars *Amphipholis squamata* (Delle Chiaje, 1828), *Ophiocomina nigra* (Abildgaard in O.F. Müller, 1789), and *Ophiothrix fragilis* (Abildgaard in O.F. Müller, 1789) as well as the holothurian *Aslia lefevrei* (Barrois, 1882) were found on the spider crab *Maja squinado* (Herbst, 1788) in the Ria de Arousa, Atlantic Ocean [29].

During our previous studies conducted in the same area and in other coastal areas of the Barents Sea [12,13] only one echinoderm species, i.e., *Ophiura robusta* (Ayres, 1852) was found on red king crabs in the Barents Sea, thus the present records expand a list of echinoderm epibionts that could settle on *P. camtschaticus*.

Among other starfish, *Asterias rubens* is the most common and familiar at shallow water sites of the North, Baltic, White, and Barents Seas. This starfish has typically five tapering arms of the same length. Usually, the aboral part of the *A. rubens* body is bright orange, pale brown, or violet while the oral part is pale yellow [30]. This species may reach 52 cm, but the majority of specimens are 10–30 cm in diameter. Spawning occurs in February-June depending on location [31]. *A. rubens* is considered to be a predator and scavenger with a wide range of food items, including bivalve mollusks, polychaete worms, other echinoderms and small crustaceans [30,32,33]. Natural predators of *A. rubens* are demersal fish, other starfishes, crabs, and lobsters [34].

*Strongylocentrotus droebachiensis* is found in Northern Europe and on the East Coast of the Canada and USA. In the North Pacific, it extends along the east coast of Siberia to the middle of the Kuril Island chain and the east coast of Sakhalin Island, and from the Aleutian Islands and Alaska down the west coast of North America to Oregon [35]. The green sea urchin is a long-living (45 years) and slow-growing species. It is an omnivorous grazer preferring brown algae, epiphytes (Hydroidea, Bryozoa, Spongia), and gastropod and bivalve mollusks, especially *Mytilus edulis* (Linnaeus, 1758) [23].

*Ophiura sarsii* is a circumpolar species found as far south in the Pacific as 35° N. It occurs in the North Barents Chukchi and western Beaufort Seas where it occurs on soft sediments to a depth of 2000 m [36]. The reddish or darker body of this species has a central disc of up to 40 mm in diameter and 5 arms extending to 90 mm or 3–4 times the disk diameter [37]. *Ophiura sarsii* is a trophic generalist feeding on arthropods, annelids, mollusks, and cnidarians. Amphipods are the most preferable food items for this species [38]. Fish, sea stars, and crabs are the major predators of brittle stars [39].

*Cucumaria frondosa* occur in the North Atlantic from the Arctic to Cape Cod and from the Arctic to the northern latitudes of the United Kingdom, in Iceland, in the North Sea (to the south of the Dogger Bank) and along the coast of Greenland, in the Barents and Norwegian Seas [40]. This species colonizes rocky or pebbly bottoms in the coastal zone of the Kola Peninsula and sandy bottoms in the open sea [41]. The maximum age of *C. frondosa* is 22 years. Sea cucumbers are mainly sessile and their main food items are detritus, pellets, mineral particles, and phytoplankton [42].

Although there are no prior reports that suggest epibiotic interactions between *P. camtschaticus* and *A. rubens, O. sarsii,* and *C. frondosa*, predator-prey relationships between these species have been well documented in the Barents Sea both by Russian and Norwegian scientists [6,15]. While in Dalnezelenetskaya Bay, bivalves and gastropods were the most important prey items for red king crabs (with a frequency of occurrence over 60%), sea urchins, sea stars, and brittle stars were also frequently consumed by red king crabs (>50% of all adults) [6].

We found higher prevalences of echinoderms on male red king crabs. This pattern is more likely associated with differences in the behavior of males and females. Males tend to migrate more frequently and cover longer distances than females [2] and, therefore, they have a higher chance to be colonized by epibionts.

Situations such as when free-living prey species infest predatory hosts to avoid death from their attacks, are rare but not unique. For example, Poulter et al. [43] have reported that the blue mussel *Mytilus edulis* can infest the branchial chamber of the shore crab *Carcinus maenas* in the English Channel and Gullmar Fjord, Sweden. A similar association was described by Villegas et al. [44] who found the rock mussel *Semimytilus algosus* on the body of the sand crab *Emerita analoga* from southern Peru.

Echinoderm colonizers may derive a variety of other benefits from red king crabs. It is known that adult red king crabs show a defense behavior, and they protect themselves against attacks from their conspecifics and other predators [45]. In addition, during their migrations, red king crabs can cover distances as long as 69 km per 90 days [46]. For these reasons, living in association with *P. camtschaticus* may be advantageous for sea stars, brittle stars, sea urchins, and sea cucumbers because it may provide them protection from predators and increases their mobility. Male crabs migrate more actively than females [1,2] and they have a higher chance to be colonized by epibionts. Our data support this pattern because only one epibiont was found on a female red king crab. Additionally, red king crabs could provide sea stars and brittle stars with food items such as mussels *Mytilus edulis*, polychaetes *Harmothoe imbricata* (Linnaeus, 1767) and *Chone* sp., copepods *Tisbe* spp. or/and amphipods *Ischyrocerus commensalis* (Chevreux, 1900) and *Ischyrocerus anguipes* (Krǿyer, 1838) which can colonize the carapaces and other parts of red king crab in large amounts [11,12,13,47]. *Cucumaria frondosa* could ingest detritus concentrated on the crab body.

While consequences of colonization by *S. droebachiensis, O. sarsii* and *C. frondosa* for *P. camtschaticus* are unknown, it seems unlikely to provide disadvantages due to the small sizes of these epibionts and their localization on the abdomen and carapace. Usually, small epibiotic species may pose a threat to their crustacean hosts if they live in the gills or on hosts’ broods causing a decrease of hosts’ gill efficiency and an increase in egg mortality and a further decrease of population density of the hosts [12,48,49,50].

On the other hand, it may be expected that the presence of *A. rubens* could have negative effects for *P. camtschaticus* owing to the increased weight, reduced mobility, and restriction of the functions of several organs such as eyes and appendages. For example, the barnacle *Amphibalanus amphitrite* (Darwin, 1854) adds weight to gastropod shells and causes their rejection by hermit crabs in Tampa Bay, Florida [50]. Other epibionts such as bryozoans and tunicates add significant weight to hermit crabs from Australia and the USA [51,52]. However, such effects occur in the case of significant weights of epibionts (more than 100% of the host weight). In our case, the sea stars added only 1.9–6.3% of the total crab weight and, therefore, most likely did not affect the mobility of their hosts. Nevertheless, the additional weight may be a problem in the case of long migrations of the hosts because of increased energy requirements. *Asterias rubens* may negatively impact their hosts by damaging soft tissues of crabs with already damaged or perforated shells. Additional studies are required to test these assumptions.

Population growth and geographical expansion of introduced marine animals and plants could lead to range expansion of their symbionts and epibionts [20]. This pattern is relevant for the red king crab population in the Barents Sea as well. For example, we recorded range expansion of the symbiotic amphipod *I. commensalis* in coastal waters of the Kola Peninsula [12,13,21,47] with no negative effects for the local bottom communities [13]. Hemmingsen et al. [53] suggested that the burgeoning population of red king crab along the coast of Finnmark (North Norway) is indirectly responsible for increasing transmission of trypanosomes *Trypanosoma murmanense* (Nikitin, 1927), parasites of crab-associated fish leeches *Johanssonia arctica*, to cod *Gadus morhua* (Linnaeus, 1758). In contrast to the amphipods and fish leeches, *A. rubens* and other echinoderm epibionts are common members of local benthic communities and they have low prevalence rates on *P. camtschaticus*, hence these epibiotic associations have no negative consequences for populations of other species and do not affect the fishery of red king crab and commercial fish in the Barents Sea.

## 5. Conclusions

This is the first report of typically free-living echinoderms *Asterias rubens*, *Ophiura sarsii*, *Strongylocentrotus droebachiensis* and *Cucumaria frondosa* colonizing the body of *Paralithodes camtschaticus* not only in the Barents Sea but also in the crab’s native areas. Because infestations are relatively rare, occurring in only 0.7–1.1 % of individuals inspected, more extensive sampling of a greater number of crabs at other sites would be necessary to accurately establish the abundance and prevalence of echinoderms on their crustacean hosts. Further studies are also necessary to fully understand the effects of these relationships and to assess ecological consequences for the host and its epibionts. The associations between echinoderms and red king crabs could have important ecological implications and provide an interesting example of how a prey species can avoid death by infesting its predator.

## Figures and Tables

**Table 1:** Intensity of epibionts found on red king cabs together with echinoderms in Dalnezelenetskaya Bay, Barents Sea in 2010, 2018, and 2019.

Other Epibionts	Echinoderm Epibiont					
	Sea Star	Sea Urchin	Sea Star	Sea Star	Brittle Star	Sea Cucumber
	2010	2010	2018	2019	2019	2019
*Ampelisca* sp.	–	–	–	–	3	–
*Balanus crenatus* Bruguière, 1789	–	2				
*Callopora lineata* (L., 1767)	–	–	+	–	+	–
*Caprella* sp.	–	–	–	–	1	–
*Chone* sp.	–	–	–	1		–
*Craniella cranium* (Müller, 1776)	–	–	–	–	1	–
*Coryne hincksi* Bonnevie, 1898	–	1	–	–	–	–
*Crisia denticulata* (Lamarck, 1816)	–	–	+	–	+	–
*Disporella hispida* (Fleming, 1828)	–	–	+	–	+	–
*Ectinosoma normani* Scott T. & A., 1896	–	–	–	4	–	–
*Eumida sanguinea* (Örsted, 1843)	–	–	–	–	1	–
*Harmothoe imbricata* (L., 1767)	–	–	–	–	2	–
*Harpacticus chelifer* (O.F. Müller, 1776)	–	–	–	–	–	1
*Ischyrocerus anguipes* Krǿyer, 1838	–	–	–	–	71	1
*Ischyrocerus commensalis* Chevreux, 1900	6	5	18	51	113	68
*Johanssonia arctica* (Johansson, 1898)	1	–	–	–	2	–
*Lacuna vincta* (Montagu, 1803)	–	–	–	–	1	–
*Mesochra pygmaea* (Claus, 1863)	–	–	–	4	–	–
*Obelia longissima* (Pallas, 1766)	–	+	–	–	+	–
*Patinella verrucaria* (Linnaeus, 1758)	–	–	–	–	+	–
*Semibalanus balanoides* (Linnaeus, 1767)	–	–	–	–	40	–
*Tisbe furcata* (Baird, 1837)	–	1	45	20	251	17
*Tisbe minor* (Scott T. & A., 1896)	–	–	–	–	–	3
*Tisbe tenera* (Sars G.O., 1905)	–	–	20		–	4
*Tricellaria arctica* (Busk, 1855)	–	+	+	–	+	–
*Zaus spinatus* (Goodsir, 1845)	–	–	–	1	–	–

Note: infestation intensities are presented for solitary species only, “+” indicates the presence of colonial epibiont species.

## Data Availability

The data are available on request from the corresponding author.
